# Sketchbook: logical model inference from Boolean network sketches

**DOI:** 10.1093/bioadv/vbag014

**Published:** 2026-01-22

**Authors:** Ondřej Huvar, Nikola Beneš, Luboš Brim, Samuel Pastva, David Šafránek

**Affiliations:** Faculty of Informatics, Masaryk University, Brno 602 00, Czech Republic; Faculty of Informatics, Masaryk University, Brno 602 00, Czech Republic; Faculty of Informatics, Masaryk University, Brno 602 00, Czech Republic; Faculty of Informatics, Masaryk University, Brno 602 00, Czech Republic; Faculty of Informatics, Masaryk University, Brno 602 00, Czech Republic

## Abstract

**Summary:**

Sketchbook is a tool for design and analysis of *Boolean network sketches*, a framework for partial specification of Boolean network models combining static and dynamic logical constraints. The tool combines an intuitive graphical interface with a high-performance inference engine able to efficiently compute the whole set of all admissible candidate models.

**Availability and implementation:**

All software and data are freely available as a reproducible artefact at https://doi.org/10.5281/zenodo.15828328. The up-to-date version of the tool is accessible through https://github.com/sybila/biodivine-sketchbook.

## 1 Introduction

Boolean networks (BNs, see e.g. Wang *et al.* 2012) represent a simple yet expressive formalism for modelling various processes in living cells. An important modelling problem is how to build the model from available information (prior knowledge, experimental data, etc.); the so-called *BN inference problem*. In Beneš *et al.* (2023), we have presented an inference method based on the concept of *Boolean network sketches*, a formalism that integrates partial knowledge about the network topology and update logic with dynamical constraints representing knowledge or assumptions about the system’s behaviour (e.g. the attractor landscape). We now present Sketchbook, a tool that allows to design BN sketches in a semi-graphical way and implements the inference method presented in Beneš *et al.* (2023).

**Figure 1:**
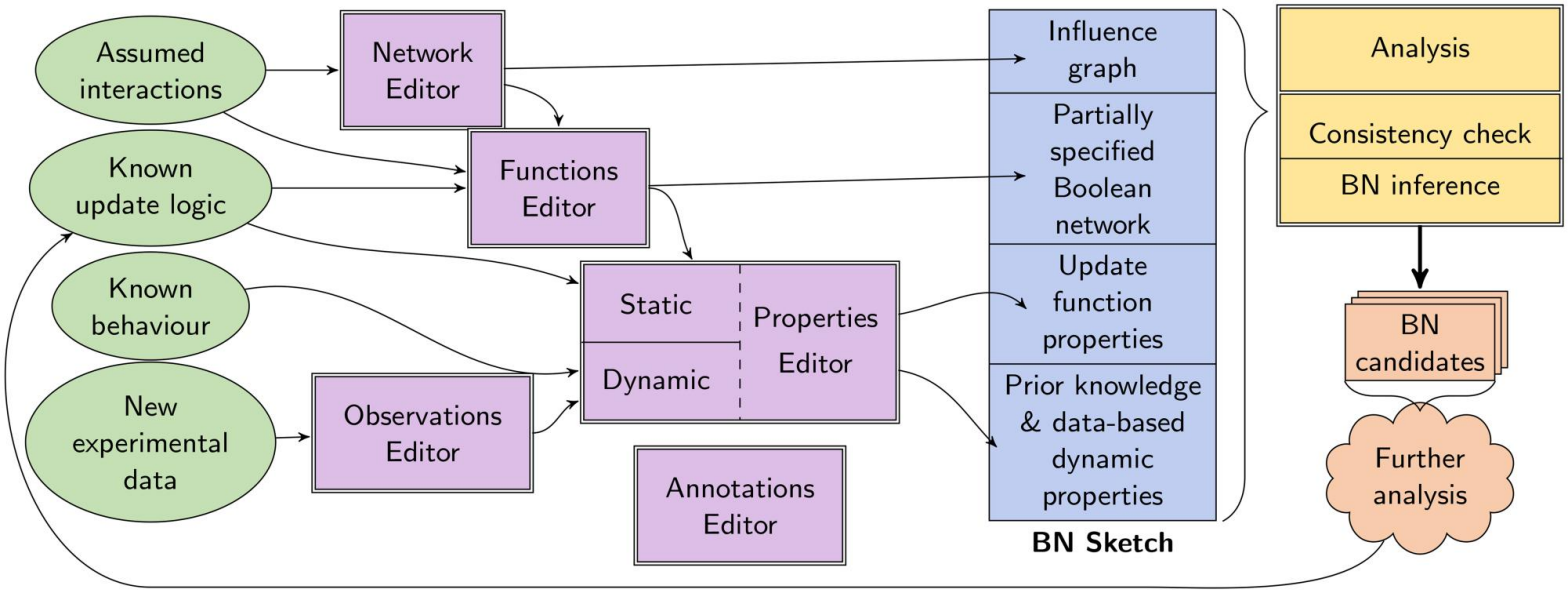
Illustration of the structure of Sketchbook and the workflow within. Elliptical nodes, highlighted in green, represent inputs: prior knowledge (or possible hypotheses) about the topological structure of the interactions, about the update logic and behaviour of the model, as well as experimental data that has been binarized. Purple, double-outlined rectangles denote the core components of the Sketchbook’s graphical editor. The blue rectangle represents the Boolean network sketch, the internal data structure used for inference. The mapping between the editor’s components and the constituent parts of a Boolean network sketch is illustrated with arrows. The yellow rectangle indicates the Sketchbook’s Analysis tab and Inference Session, responsible for consistency checks and the BN inference computation. Note that the resulting set of Boolean network candidates can be further processed to obtain new knowledge (or new hypotheses) which can then be used to refine the original input.

A Boolean network sketch consists of several parts, as depicted in blue on the right-hand side of Fig. 1. The first part is an *influence graph*, which describes the topology of the modelled network. Formally, it is a binary relation that consists of all the possible *regulations* between the variables of the BN. The second part, a *partially specified Boolean network* (PSBN), builds on the influence graph by assigning each variable an expression that determines how it is updated. Unlike standard BNs, these expressions may contain function symbols representing unknown parts of the update mechanism to be determined by the inference method. By substituting concrete Boolean functions in place of the function symbols (called an *interpretation*) we obtain a standard BN. All the BNs obtainable by this mechanism are called *consistent* with the PSBN.

The third part of the sketch reflects further prior knowledge about the update mechanism of the BN variables and restricts the allowed interpretations. We call this part the *update function properties*. Formally, these properties are defined in the first-order logic over Booleans. The logic allows us to describe regulations as essential (i.e. having an effect in all BNs), positively or negatively monotone (i.e. activations or inhibitions), and much more; for detailed examples see the Supplementary Material of Beneš *et al.* (2023).

The last part of the sketch represents (prior or data-based) knowledge about the dynamic behaviour of the system to be modelled. These *dynamic properties* are formally expressed in the hybrid extension of Computation Tree Logic (CTL), called HCTL (Lange 2009). HCTL allows reasoning about the system’s long-term and transient dynamics, such as attractors and their relationships, complex periodic behaviour, stabilities, etc. Common HCTL properties can be automatically derived by Sketchbook from experimental observations. Here, the observations can be of steady state or time-series behaviour. However, we assume the input data is already binarized by some preexisting tool. Typically, the data come from transcriptomics (gene expression), but any data assessing the activity of the BN variables can be used. A detailed description of the syntax, semantics, and example properties of HCTL can be found in the Supplementary Material of Beneš *et al.* (2023).

The Sketchbook tool allows users to create, manipulate, and analyse Boolean network sketches via a graphical interface. The goal of the analysis is to obtain the set of all BNs consistent with the sketch, thus providing a *logical* approach to the BN inference problem. The results are produced in a compact representation of the whole set and can be used for further processing. We give a brief overview of the tool’s implementation and describe the various components from user perspective in the Tool structure section. Following that, in Section 3, we demonstrate the applicability of the tool on a short case study, including a comparison with related tools (see below). Further details about the tool, including the screenshots and evaluation results, are given in the Supplementary Material, available as supplementary data at *Bioinformatics Advances* online.

### 1.1 Related tools

In terms of logical approaches to BN inference, the closest related tools are Griffin (Muñoz *et al.* 2018), BoNesis (Chevalier et al. 2024), RE: IN (Yordanov *et al.* 2016) and its reimplementation BRE: IN (Goldfeder and Kugler 2019). The main differences between these tools and Sketchbook are (i) none of these tools offer an interactive graphical user interface allowing easy manipulation of the network, (ii) all of these tools employ enumerative methods, producing the candidate networks one at a time, while Sketchbook builds a symbolic representation of the whole candidate set (this allows, for example, true *uniform* random sampling of solutions, even if the solution space is too large to enumerate); (iii) the dynamic properties dealt with by these tools are more limited compared to the full expressive power of HCTL utilized by Sketchbook; and (iv) the tools typically restrict the possible shapes of the update functions to a fixed set of options. Note that BoNesis allows arbitrary update functions, but provides the user with the possibility to limit the number of DNF clauses, which is necessary to deal with large in-degree influence graphs.

The tools also differ with respect to the Boolean network semantics. Griffin uses synchronous semantics only, RE: IN and BRE: IN allow to choose between synchronous and fully asynchronous semantics, and BoNesis uses the so-called most-permissive semantics (Paulevé *et al.* 2020). Our tool currently only supports the fully asynchronous semantics. Despite the difference in semantics, we give at least some insight into the tools’ differences performance-wise in Section 3 by providing a comparison on a case that lies within a common fragment of the tools’ inputs.

In addition to the logic-based approaches, there also exists a distinct group of methods based on heuristic optimization techniques, recently reviewed in Pušnik *et al.* (2022). While often effective in fitting experimental data, they generally produce only a single candidate or a small subset of plausible networks and can lack formal guarantees regarding the completeness or stability of the inferred solutions.

## 2 Tool structure

The functionality of Sketchbook is divided into two main parts. The *Editor Window* enables users to design and edit all parts of a BN sketch in a user-friendly manner. The subsequent *Inference Session* is responsible for the inference computation and allows users to examine the results. The tool combines a Rust back end for intensive computations with a TypeScript-based front end for the user interface, integrated into a unified desktop application.

**Figure 2:**
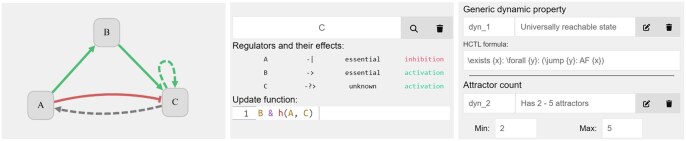
Screenshot snippets of selected components of the tool. The left-most snippet showcases the graph-based interface of the Network Editor on a small model of three variables. The style and colour of the arrows illustrates properties of the regulations—green arrows are activations, red are inhibitions, and dashed regulations are non-essential. The middle snippet displays the Functions Editor, showing the regulators and update function of the variable *C*. The right-most snippet shows examples of two dynamic properties (part of the Properties Editor). The first is a user-defined property written in HCTL stating that there has to exist a universally reachable state. The second property is created using one of our templates, stating the desired attractor count.

The main workflow of the tool and its various components are illustrated in Fig. 1. Importantly, there is a mapping of how the components of the editor correspond to the formal definition of a Boolean network sketch. We also provide screenshot snippets of a few selected components of the tool in Fig. 2, with a more extensive collection of screenshots provided throughout Section 2 of the Supplementary Material, available as supplementary data at *Bioinformatics Advances* online.

The main entry point for the user when creating a new sketch is the *Network Editor*. This editor provides a GUI to manipulate the influence graph by adding, removing, and editing variables (graph nodes) and regulations (graph edges, i.e. arrows). For convenience, the editor also allows to set some update function properties in terms of essentiality and monotonicity of regulations. These are depicted visually by the colour and style of the arrow lines.

The expressions representing the update functions of a PSBN are dealt with in the *Functions Editor*. Here, the user can also create new function symbols representing unknown parts of the update mechanism and use them in the update expressions for the individual variables. Note that if no update expression is explicitly provided by the user for a variable, the tool automatically creates a fresh function symbol for that variable and sets its update expression to be this function symbol applied to all its regulators. The Functions Editor also includes some of the update function properties, namely those that can also be set in the Network Editor.

A more fine-grained manipulation of the update function properties is provided by the *Properties Editor*, more specifically its first part, the *Static Properties Editor*. The user may either choose one of predefined property templates or provide own properties by writing them as first-order logic formulas. For completeness, the Static Properties Editor also lists all the update function properties provided by the user in the previous two editor tabs.

The second part, the *Dynamic Properties Editor*, deals with the dynamic behaviour of the modelled system. The user can either write their own HCTL formulas or select one of the pre-defined dynamic property templates, such as the number of attractors. Some of the templates work with datasets of binarized experimental data, such as time-series measurements or steady-state observations. These datasets can be imported and manipulated through the *Observations Editor*.

Users can also annotate all important elements, such as variables, observations, properties, and more. These annotations are displayed in a structured way in the *Annotations Editor*. Moreover, Sketchbook enables the user to import models in standard formats like AEON or SBML (as well as an internal JSON format). Once the sketch is fully prepared, the user can start the Inference Session from the *Analysis* tab and run the process of BN inference.

Sketchbook then converts all the inputs into internal structures and executes the main BN inference algorithm, computing the set of *all* candidate BNs consistent with the sketch. More details on the algorithm are provided in Section 2.2 of the Supplementary Material, available as supplementary data at *Bioinformatics Advances* online. The computation runs completely asynchronously, ensuring a non-blocking execution that allows users to continue working in the editor or run multiple parallel computations.

After the inference computation is finished, the tool shows the user a report summarizing the computation, and the user can sample individual candidate Boolean networks. The tool also displays the number of possible update function variants for each variable across the whole candidate set. In addition, the user can also directly export a compact symbolic representation of the entire set of candidates to use for further analysis, for instance, using the *AEON.py* library (Beneš *et al.* 2022).

## 3 Results

In this section, we evaluate the performance and practical use of Sketchbook. First, we showcase the tool on a small case study and compare it with other relevant BN inference tools. Next, we test the tool’s performance on a set of real-life biological networks with synthetic steady-state data. All experiments have been performed on a standard workstation with an 11th Gen Intel i5 CPU and 16 GB RAM.

In La Rota *et al.* (2011), a partially specified model with 21 variables was created for a signalling network governing sepal development in *Arabidopsis thaliana*. The network includes 19 hypothetical regulations, leading to a wide range of candidate models. Additionally, expected steady-state data were constructed by examining expression patterns of key genes essential for sepal development. We use this network and data as a demonstration of Sketchbook and to compare its performance to the three previously mentioned inference tools—Griffin, BoNesis, and BRE: IN.

To ensure compatibility, we restrict the input to a common subset of properties supported by all the tools. The steady-state observations are interpreted as required fixed-point attractors, which all three tools can process. Fixed-point attractors are also preserved across different Boolean network semantics, enabling direct comparison. It is worth noting that Sketchbook is designed to handle far more complex dynamic properties. Similarly, we also limit the update function properties to only the fragment supported by all tools.

To execute the experiment with Sketchbook, we first use the Network Editor to construct the influence graph and select regulation types according to the information provided in La Rota *et al.* (2011). Sketchbook automatically derives the set of update function properties, capturing monotonicity and essentiality of regulations. We then load the binarized steady-state dataset into Observations Editor. In the Properties Editor, we create a new dynamic property using the *fixed points* template and select the dataset. In the Analysis Tab, we simply start the Inference Session and then run the inference. Sketchbook computes the set of all 439 296 BNs consistent with the input sketch in about 1 s.

Next, we used the same network and properties to run the inference with BoNesis, Griffin, and BRE: IN. BoNesis completed its computation in 17 s, while Griffin took over 2 h. Both tools identified the same number of 439 296 candidate models. Note that the Zenodo artefact contains more benchmark tests on models acceptable by BoNesis. The candidates identified by BoNesis and Sketchbook match exactly, thus further validating our implementation. When evaluating BRE: IN, we encountered considerable issues. First, the tool only generates a single Boolean network for each variant of the regulatory graph, omitting other valid update logic settings (even though thousands of consistent BNs may exist for a single graph). Second, BRE: IN was unable to complete the enumeration beyond the first 120 solutions, repeatedly terminating with an error. The time taken to enumerate these first 120 solutions was 10 s.

Even in this simple scenario designed to align with the capabilities of the other tools, Sketchbook demonstrates a substantial performance advantage over Griffin and BRE: IN, achieving approximately a 1000-fold speed increase (noting that BRE: IN was unable to complete the computation). BoNesis, while significantly faster than Griffin and BRE: IN, employs an enumeration approach that might affect its efficiency when dealing with a large number of solutions. We give a more detailed explanation in Section 3.1 of the Supplementary Material, available as supplementary data at *Bioinformatics Advances* online.

By further examining the candidate models, we found that some of them had extra attractors not seen in our steady-state data. To refine the results, we added a dynamic property to exclude all additional attractors. Even with this additional property, Sketchbook completed the computation in under 2 s. This refinement reduced the number of consistent candidates to 48 352, approximately a 10-fold decrease. Moreover, we also used Sketchbook to verify that all the remaining models have only fixed points and no complex attractors.

Furthermore, Sketchbook provides additional deeper insights into the inference results. The tool’s summary of the candidate set reveals that all consistent candidates agree on the update functions for 11 variables. This information can help modellers refine the sketch. By integrating these confirmed update functions, they can narrow their focus on the 10 remaining network variables, whose update functions still vary among the candidates.

Finally, we evaluated Sketchbook’s performance on a set of complex sketches based on real-world models and synthetically generated steady-state data. The tool successfully performed inference on PSBNs with up to 321 variables and more than $2^{100}$ interpretations. The results are summarized in Table 1. Notably, even for the largest models, computation times did not exceed 10 min. Additional details can be found in Section 3 of the Supplementary Material, available as supplementary data at *Bioinformatics Advances* online.

**Table 1:** Performance evaluation of Sketchbook on a set of partially specified models of various sizes (see Section 3).^a^

Model name	Number of variables	Number of BNs consistent with PSBN	Number of BNs consistent with whole sketch	Sketchbook computation time (s)
Cell Div B	9	4.1e6	1.4e4	0.2
E Protein	35	1.8e22	1008	2.5
NSP4	60	7.5e22	128	2.1
ETC	84	4.7e21	3.1e6	370.1
Interferon I	121	1.0e31	6.8e5	170.7
NSP9	252	8.5e37	5.5e10	28.3
Macrophage	321	2.0e31	7.8e11	332.2

a The first two columns provide the model’s name and variables count. The third column shows the number of BNs consistent with the input PSBN, i.e. the size of the initial solution space before considering any static or dynamic properties. The fourth column then shows the number of BNs consistent with the whole sketch (i.e. the set of all BNs that satisfy the entire specification, which is returned by the tool). The last column reports the computation time in seconds, which includes every step from reading the input to generating the entire set of all admissible solutions.

## Supplementary Material

vbag014_Supplementary_Data

## Data Availability

All software and data are freely available as a reproducible artefact at https://doi.org/10.5281/zenodo.15828328. The up-to-date version of the tool is accessible through https://github.com/sybila/biodivine-sketchbook.
