# Supplementary material for: Sketchbook: logical model inference from Boolean network sketches
Source: Bioinform Adv. 2026 Jan 22;6(1):vbag014. doi: 10.1093/bioadv/vbag014 (PMC12883443; doi:10.1093/bioadv/vbag014)
Supplement: vbag014_Supplementary_Data [file vbag014_supplementary_data.pdf]

SKETCHBOOK: Logical Model Inference from Boolean Network Sketches (Supplementary Material)

This document extends the main article with the following information. Section 1 provides relevant theoretical background, formally introducing Boolean networks (BNs) and BN sketches. In Section 2, we give details regarding the tool's design and implementation, including the architecture, main BN inference algorithm, and user interface. Lastly, Section 3 expands the performance evaluation briefly discussed at the end of the main article.

1 Preliminaries

1.1 Boolean Networks

We use $\mathbb{B} = \{0, 1\}$ to denote the set of Boolean values in the following. A *Boolean network* $F^{(n)}$ consists of n Boolean variables $v_1, ..., v_n$. We denote this set of variables Var_n. Each variable v_i is equipped with a logical *update function* $F_i : \mathbb{B}^n \to \mathbb{B}$ that dictates how the variable evolves. For a function F_i, its dependency set $dep(F_i) \subseteq Var_n$ is the set of network variables that actually influence the output of F_i. Note that in our notation, we sometimes omit the arity of the BN when clear or not needed, simply writing F instead of $F^{(n)}$. We do this for other structures defined in the following text as well.

There are different ways how to define the semantics of BNs, depending on a chosen *update mode*. In synchronous update mode, all variables are updated simultaneously at every time step. On the other hand, in asynchronous update mode, only a single variable is updated in each time step. We consider asynchronous semantics in our work, as it is closer to the biological reality, where individual reactions happen in different time scales. The dynamics of an asynchronous BN can be represented as a non-deterministic state-transition graph (STG). A state of a BN with n variables is a Boolean-valued vector of length n, assigning a binary value to each variable. The corresponding state space $S = \mathbb{B}^n$ is thus a set of all Boolean-valued vectors of length n. The transition relation $T \subseteq S \times S$ is defined as follows:

$$(s, t) \in T \Leftrightarrow s \neq t \wedge \exists i \in \{1, \ldots, n\}.\ t = s[i \mapsto F_i(s)]$$

Here, $s[i \mapsto F_i(s)]$ denotes the copy of state s where the value of the i-th element is updated to the value given by $F_i(s)$. This means a single (i-th) variable gets updated according to its update function evaluated at the given state. For technical reasons, we also consider self-loops for "sink" states without successors.

1.2 BN Sketches

A Boolean network sketch consists of four components: an influence graph, a partially specified Boolean network, a set of update function properties and a set of dynamic properties.

1.2.1 Influence Graph

An *influence graph* (IG) specifies the *possible dependencies* between individual variables. More formally, given a set Var_n of n Boolean variables, the *influence graph* is a binary relation $I^{(n)} \subseteq \mathrm{Var}_n \times \mathrm{Var}_n$.

We say that a particular Boolean network F is *consistent* with IG I if for every F_i and every $v_j \in \mathit{dep}(F_i)$, we have that $(v_j, v_i) \in I$. In other words, all variables influencing the output of a variable's update function must be among its regulators in the influence graph. Note that the opposite (i.e. $(v_j, v_i) \in I \Rightarrow v_j \in \mathit{dep}(F_i)$) is not required; the IG can be seen as an upper bound on possible regulations.

1.2.2 Partially Specified Boolean Network

Partially specified Boolean networks (PSBNs) allow representing partial (incomplete) knowledge regarding the rules governing the network's dynamics. A PSBN extends the concept of Boolean networks by incorporating *function symbols*, thus allowing for partially specified update functions. Formally, we assume a set $\mathcal{F}$ of function symbols with specified arities. We use the notation $\boldsymbol{g}^{(a)}$ for function symbols (where $\boldsymbol{g}$ is the function symbol and a its arity). A PSBN $E^{(n)}$ consists of n Boolean variables, where each variable v_i is equipped with a partially specified Boolean expression E_i. The expressions are defined by the following grammar (v ranges over the Boolean variables):

$$E ::= 0 \mid 1 \mid v \mid \neg E \mid E \wedge E \mid \boldsymbol{g}^{(a)}(E, \ldots, E)$$

As usual, we can define derived Boolean operators such as disjunction ($\vee$), exclusive disjunction ($\oplus$), implication ($\Rightarrow$), etc.

An *interpretation of* $\mathcal{F}$ is a function $\mathcal{I}$ that maps each function symbol $\boldsymbol{g}^{(a)} \in \mathcal{F}$ to a Boolean function of the same arity. By selecting a particular interpretation $\mathcal{I}$ for a given PSBN E, we obtain a concrete Boolean network (an instance of the PSBN, which we denote by $E(\mathcal{I})$). A BN F is *consistent* with a PSBN E if there exists an interpretation $\mathcal{I}$ such that $F = E(\mathcal{I})$.

1.2.3 Update Functions Properties

We often require that update functions satisfy certain properties, thus limiting the considered interpretations. For example, we might require a particular update function to be positively or negatively monotonic with respect to a specific input variable. This corresponds to the notion of activating, resp. inhibiting regulation. We might also require that a regulation have an "observable effect" by expressing that a particular input of the corresponding update function is *essential* (i.e., influencing the output of the function).

To express these kinds of properties, we use the first-order logic (FOL) over Booleans. We use $\mathbb{F}^{(n)}$ to denote the set of all Boolean functions of type $\mathbb{B}^n \to \mathbb{B}$ and define a *Boolean function property* to be a FOL-definable predicate over $\mathbb{F}^{(n)}$. To show a few examples, consider:

- ith input is essential in function g:
  $essential_i(g) := \exists x \in \mathbb{B}^n.g(x[i \mapsto 0]) \oplus g(x[i \mapsto 1])$;
- function g is positively monotonic in its ith input:
  $positive_i(g) := \forall x \in \mathbb{B}^n.g(x[i \mapsto 0]) \Rightarrow g(x[i \mapsto 1])$;
- function g is negatively monotonic in its ith input:
  $negative_i(g) := \forall x \in \mathbb{B}^n.g(x[i \mapsto 1]) \Rightarrow g(x[i \mapsto 0])$.
- function g is non-monotonic (*dual*) in its ith input:
  $dual_i(g) := \neg positive_i(g) \wedge \neg negative_i(g)$.

More examples can be found in the Supplementary material of [1].

By evaluating the properties over the update functions of a classical BN, we get a yes/no answer, i.e. $essential_i(F_j)$ is true if and only if $v_i \in dep(F_j)$ and so on. We extend this evaluation to partially specified Boolean expressions. We denote this by $prop[E_j]$, where $prop$ is a property and E_j is a partially specified Boolean expression. The result of evaluating a property over a partially specified expression is the set of all interpretations for which the resulting Boolean function satisfies the property. For example, $positive_i[E_j]$ would evaluate to a set of all interpretations that ensure that (v_i, v_j) is an activation regulation. Formally, property evaluation is defined as $prop[E] = \{\mathcal{I} \mid prop(E(\mathcal{I})) \text{ is true}\}$.

The *update function properties* for a PSBN are given as a set Π of Boolean function properties applied to partially specified Boolean expressions that constitute the PSBN. Semantically, Π corresponds to the set of all interpretations that satisfy each $\pi \in \Pi$, i.e. the set intersection of all the property evaluations. We say that an interpretation $\mathcal{I}$ is *consistent* with Π if it belongs to this set. We further say that a BN $F^{(n)}$ is *consistent* with both a partially specified BN $E^{(n)}$ and its update function properties Π if there exists an interpretation $\mathcal{I}$ consistent with Π such that $F = E(\mathcal{I})$.

1.2.4 Dynamic Properties

Finally, we also want to express properties about the behaviour of the model, i.e. properties of the corresponding STG. To do so, we employ a hybrid extension

of the Computation Tree Logic called HCTL. The following presentation of the HCTL follows [2].

Definition 1. *Let* AP *be a finite set of* atomic propositions *and* Var_{s} *be a countable set of* state variables. *A* dynamic property φ *is a HCTL formula defined as follows (*p *ranges over* AP *and* x *over* Var_{s}*):*

$$\varphi ::= p \mid x \mid \neg\varphi \mid \varphi \wedge \varphi \mid @_x\ \varphi \mid \downarrow\! x.\ \varphi \mid \exists x.\ \varphi \mid \mathbf{EX}\,\varphi \mid \mathbf{E}[\varphi\,\mathbf{U}\,\varphi] \mid \mathbf{A}[\varphi\,\mathbf{U}\,\varphi]$$

Before we define the formal meaning of each operator, let us also observe that we use the standard abbreviations that define additional commonly used operators:

$$\begin{aligned}
\mathbf{AX}\,\varphi &\equiv \neg\,\mathbf{EX}\,\neg\varphi \\
\mathbf{EF}\,\varphi &\equiv \mathbf{E}[\mathit{true}\,\mathbf{U}\,\varphi] \\
\mathbf{AF}\,\varphi &\equiv \mathbf{A}[\mathit{true}\,\mathbf{U}\,\varphi] \\
\mathbf{EG}\,\varphi &\equiv \neg\,\mathbf{AF}\,\neg\varphi \\
\mathbf{AG}\,\varphi &\equiv \neg\,\mathbf{EF}\,\neg\varphi \\
\forall x.\ \varphi &\equiv \neg\exists x.\ \neg\varphi
\end{aligned}$$

As before, we also use the usual propositional operators: disjunction, implication, etc.

Semantics The validity of a dynamic property relates to the state-transition graph STG(F), not the BN F directly. Consequently, we define a *run* π to be a maximal sequence of states $\pi = \pi_1, \pi_2, \ldots$ satisfying $\pi_i \rightarrow \pi_{i+1}$ for every i. Note that every maximal run is necessarily infinite, as every state in STG(F) has at least one outgoing transition.

Additionally, we extend the state-transition graph STG(F) with a labelling function $L : \mathbb{B}^n \rightarrow 2^{AP}$ which assigns to each state a subset of atomic propositions that hold in said state. For the purposes of this paper, we consider that $AP = \{p_1, \ldots, p_n\}$ contains propositions that identify states where individual variables hold. That is, $p_i \in L(s)$ if and only if v_i is true in state s. However, additional biologically motivated propositions can be also included, for example identifying states corresponding to specific phenotypes.

We write $\mathcal{R}_{F,s}$ to denote the set of all runs of STG(F) starting in s (i.e. $\pi_1 = s$), $\nu : \mathrm{Var}_{\mathsf{s}} \rightarrow \mathbb{B}^n$ to denote a valuation of the state variables, and define the

satisfaction relation of a formula φ in a given state $s \in \mathbb{B}^n$ as follows:

$$
\begin{aligned}
(F,s,\nu) &\models p && \iff p \in L(s)\\
(F,s,\nu) &\models x && \iff \nu(x) = s\\
(F,s,\nu) &\models \neg\varphi && \iff (F,s,\nu) \not\models \varphi\\
(F,s,\nu) &\models \varphi_1 \wedge \varphi_2 && \iff (F,s,\nu) \models \varphi_1 \text{ and } (F,s,\nu) \models \varphi_2\\
(F,s,\nu) &\models @_x\ \varphi && \iff (F,\nu(x),\nu) \models \varphi\\
(F,s,\nu) &\models {\downarrow}x.\ \varphi && \iff (F,s,\nu[x \mapsto s]) \models \varphi\\
(F,s,\nu) &\models \exists x.\ \varphi && \iff (F,s,\nu[x \mapsto s']) \models \varphi \text{ for some } s' \in \mathbb{B}^n\\
(F,s,\nu) &\models \mathbf{EX}\,\varphi && \iff (F,\pi_1,\nu) \models \varphi \text{ for some } \pi \in \Pi_{F,s}\\
(F,s,\nu) &\models \mathbf{E}[\varphi_1\,\mathbf{U}\,\varphi_2] && \iff \text{there exists } \pi \in \Pi_{F,s} \text{ and } i \in \mathbb{N} \text{ such that}\\
& && \qquad (F,\pi_i,\nu) \models \varphi_2 \text{ and } \forall j < i.\ (F,\pi_j,\nu) \models \varphi_1\\
(F,s,\nu) &\models \mathbf{A}[\varphi_1\,\mathbf{U}\,\varphi_2] && \iff \text{for all } \pi \in \Pi_{F,s} \text{ there exists } i \in \mathbb{N} \text{ such that}\\
& && \qquad (F,\pi_i,\nu) \models \varphi_2 \text{ and } \forall j < i.\ (F,\pi_j,\nu) \models \varphi_1
\end{aligned}
$$

As before, $\nu[x \mapsto s]$ denotes a copy of the valuation ν where the variable x is mapped to the state s. When φ is closed (i.e. without free variables), the choice of ν is irrelevant and we may simply write $(F,s) \models \varphi$.

The Supplementary material of [1] includes a collection of examples demonstrating how biologically relevant properties and experimental data can be encoded in HCTL.

Consistency Given a *closed* HCTL formula φ describing a dynamic property as outlined above, we say that a Boolean network F is *consistent* with φ if we have $(F,s) \models \varphi$ for every $s \in \mathbb{B}^n$, i.e. all the states of STG(F) have to satisfy φ. Note that this comes without loss of generality—if we instead wanted to specify that there exists at least one state satisfying a given formula φ, we can rewrite the formula to $\exists x.\ @_x\ \varphi$. This new formula holds in all states if and only if the original formula φ holds in at least one state.

1.2.5 BN Sketch Inference

Finally, a *Boolean network sketch* is the tuple $\mathcal{S}^{(n)} = (I^{(n)}, E^{(n)}, \Pi, \Omega)$, where $I^{(n)}$ is an influence graph, $E^{(n)}$ is a PSBN, Π is the set of update functions properties for $E^{(n)}$, and Ω is a set of closed HCTL formulas. We say that a Boolean network F is consistent with the sketch $\mathcal{S}$ if it is consistent with all its parts as described above. The problem of *BN Sketch Inference* is to compute the set of all BNs consistent with the given $\mathcal{S}$.

2 Tool Design

This section delves into the design of SKETCHBOOK, from the algorithms to the tool's architecture to the tool's user interface. The description is based on

the master's thesis [3], in which a preliminary implementation of the tool was created.

2.1 Overview

Sketchbook is built to address two key aspects of the workflow illustrated in Fig. 1 of the main article. The first is enabling users to construct all components of a BN sketch in a user-friendly way. The second is performing the BN inference to compute the set of all candidate BNs consistent with the sketch.

Achieving these goals involves several challenges. Central to the tool is the inference algorithm. This algorithm must be fully symbolic to efficiently handle the large space of candidate networks and be feasible to implement effectively. Supporting this, Sketchbook requires a robust back end to manage the complex data structures representing BN sketches and to perform computationally intensive tasks. The user interface must complement these capabilities, allowing users to define sketches and execute inference with ease.

We structure the rest of this section in the following way. We begin by presenting the design of the inference algorithm. Then, we outline the high-level architecture of the tool, highlighting the separation of back-end and front-end components and the organization into logical sessions to ensure modularity and flexibility. Finally, we delve into the design and workflow of the two main sessions: the editor session, which is responsible for sketch creation and editing, and the inference session, which performs inference and presents the results.

Note that an early version of the sketch editor front end for Sketchbook was established as part of the master thesis by Petr Ivičič [4]. You can find more details on the front-end design there. On the back-end side, the Sketchbook tool is partially based on our previous tools AEON [5] and BNClassifier [6]. Both of these tools deal with partially specified Boolean networks and classify the concrete interpretations according to specified criteria; AEON's classification is based on the number and structure of attractors in the system, while BNClassifier is based on a set of hybrid temporal logic properties formulated in HCTL.

2.2 BN Inference Algorithm

Building on our symbolic approach introduced in [1], we design an improved version of the BN inference algorithm tailored for practical implementation in Sketchbook.

Our high-level inference procedure, outlined in Algorithm 1, ensures modularity by breaking the inference process into distinct phases. The algorithm employs BDDs to symbolically encode and iteratively refine the set of candidate networks. It systematically narrows the large initial set of networks down into the set of BNs consistent with the given sketch.

In the beginning (*line 1*), we validate that all components of the sketch are consistent. That involves verifying that the PSBN E is in agreement with the influence graph I, that the formulas are syntactically correct, and so on. This check is done on the syntactic level.

Algorithm 1: High-level BN inference procedure.

Input: a BN sketch $\mathcal{S} = (I, E, \Pi, \Omega)$
Output: a BDD C encoding a set of candidate BNs
1 **if** $\mathcal{S}$ **is inconsistent then reject**;
2 $C \leftarrow$ BddEncodeInterpretations(E);
3 $C \leftarrow$ EnrichBddWithFolVars(C, Π);
4 **foreach** $\pi \in \Pi$ **do**
5 $\quad C \leftarrow$ EvaluateStatic(π, C);
6 $\quad$ **if** C **is unsat then reject**;
7 $C \leftarrow$ EnrichBddWithHctlVars(C, Ω);
8 $G \leftarrow$ ColouredSTG(E);
9 **foreach** $\omega \in \Omega$ **do**
10 $\quad C \leftarrow$ ColouredModelChecking($\omega, G, \mathbb{B}^n \times C$);
11 $\quad$ **if** C **is unsat then reject**;
12 **return** C;

In the next step (*line 2*), we symbolically encode all satisfying interpretations of the PSBN E as a set C. This involves encoding interpretations of all function symbols with a BDD. Each symbol $\boldsymbol{g}^{(a)}$ is substituted with a logically equivalent expression using 2^a fresh, zero-arity symbols (Boolean constants) that together encode the truth table of the original function $\boldsymbol{g}$. These Boolean constants are used to construct the BDD.

Before we can evaluate the static properties expressed in FOL, we must "enrich" the BDD with additional constants (*line 3*). These additional BDD constants are used to encode the assignments to the first-order variables occurring in FOL formulas. With that, we are able to perform a bottom-up evaluation of each static property.

Next (*lines 4, 5, 6*), we evaluate the properties of Π over the symbolic structures from the previous step. Note that the properties are expressed in the first-order logic over Booleans, and the symbolic representation involves only Boolean constants. Therefore, all the logical operations and quantifications can be implemented as BDD transformations. We gradually refine the set C by repeatedly calling the function `EvaluateStatic`. Here, `EvaluateStatic(`π`,` C`)` computes the subset of C satisfying the static property π. In the end, the set C only contains the interpretations of E consistent with all properties from Π.

At this point, C encodes a set of BNs that are consistent with the provided I, E, and Π. Before evaluating dynamic properties, we must again enrich the BDD with a different set of Boolean constants (*line 7*). This time, the set of additional BDD constants encodes the assignments to the state variables occurring in HCTL formulas. Again, this step is needed to perform a bottom-up model checking, which is discussed in the following steps.

We then create a *coloured* symbolic STG G that collectively encodes STGs corresponding to all the BNs within C (*line 8*). We have previously developed

a novel coloured model-checking algorithm for HCTL, presented in [7]. By iteratively running this bottom-up algorithm on G (*lines 9, 10, 11*), we can gradually compute the subset of interpretations that satisfy all HCTL formulas in Ω.

Finally, with *line 12*, the algorithm returns the set C with all candidates consistent with the whole sketch $\mathcal{S}$. Note that C is still symbolically represented, allowing us to keep track of very large sets. Given C, we can then easily sample individual candidate networks or use the symbolic representation for further computation.

If C becomes empty at any point of the computation, the sketch is not realisable (satisfiable). In that case, we can track the point during the computation where we encountered the empty candidate set. This gives an inclusion-minimal set of "requirements" (the PSBN and the properties evaluated up to that point), which can not be satisfied at the same time.

Algorithm 1 forms the backbone of our inference computation. Note that the individual steps of the algorithm often involve complex internal procedures. At the implementation level, SKETCHBOOK also employs additional optimizations to speed up the evaluation. For instance, attractor detection algorithms from AEON [5, 8] are used to efficiently evaluate certain attractor-related properties.

2.3 Architecture

The tool's functionality is organized into logical *sessions*, each representing a self-contained workspace. A session encapsulates its own data, state, and functionality, enabling modular and independent workflows. SKETCHBOOK currently supports two types of sessions: an *editor session*, and an *inference session*. The design is intentionally flexible, allowing additional session types to be introduced in the future.

Figure 1 illustrates the high-level workflow in SKETCHBOOK and the core functionality of each session. The editor session enables editing all components of the sketch. It allows the import and export of models and the loading of datasets. We describe it in detail in Section 2.4. Once the sketch is prepared, it can be passed to the inference session, where users can run the inference algorithms. After the inference computation is finished, users can sample candidate Boolean networks or export the symbolic representation of the whole candidate set. We describe the design of the inference session more in Section 2.5.

The tool starts with a primary editor session. New sessions are created dynamically during runtime, allowing users to manage multiple concurrent workspaces. Each session operates independently, maintaining its own lifecycle and internal state.

2.3.1 Back End and Front End

Each session can be associated with one or more front-end *windows* that provide an interface to users. These UI windows are managed separately from the session's main state, dividing each session's functionality into back-end and

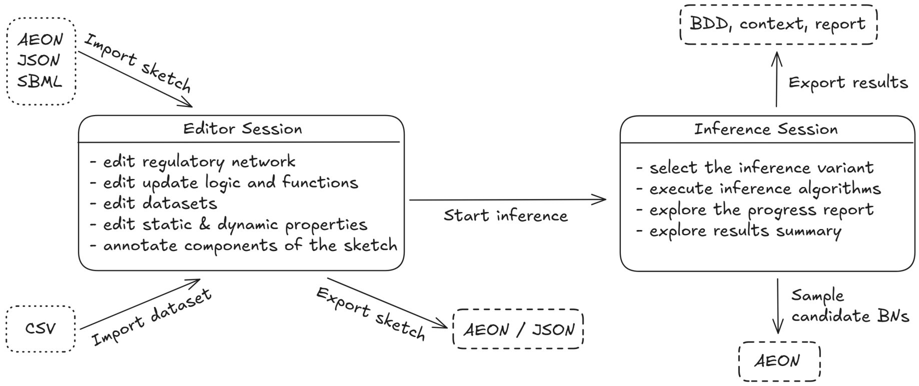

Figure 1: Illustration of the high-level workflow of SKETCHBOOK and its two main sessions.

front-end components. However, both parts are tightly synchronized. We discuss the details of this synchronization in Section 2.3.2. The back-end component is responsible for state management, intensive computations, input model parsing, and most input validations. The front end focuses on the visual aspects of the application, rendering the user interface and handling user interactions.

This modular design separates the application's logic from the user interface, improving maintainability, scalability, and ease of development. It also allows each component to use technologies best suited to its needs. For the back end, we use Rust due to its performance and memory safety, which are essential for computationally intensive inference algorithms. Using Rust also enables seamless integration with the Biodivine Rust libraries for symbolic BN analysis and model checking. For the front end, we employ a TypeScript-based stack, which offers strong typing and maintainability. Integration between the back end and front end is handled by the Tauri framework [9], which supports modular development and facilitates responsive event-driven communication.

The front-end and back-end parts communicate through custom session-specific events described in Section 2.3.2. This scoped communication ensures seamless operation and prevents interference between sessions.

2.3.2 Event-Based Communication

The communication between the session's back end and its front-end windows is realized through exchanging event messages. The action of the user (e.g., clicking on a button) is registered on the front end and is transferred to the back end via a *user action* event. When it gets to process the event, the back end validates the data, performs requested changes to the state, and sends a *state-change* event back to the front end. Once the front end gets to this event, it simply updates its data based on the changes that have been received. An example of this event exchange (triggered by a user modifying a variable's update function)

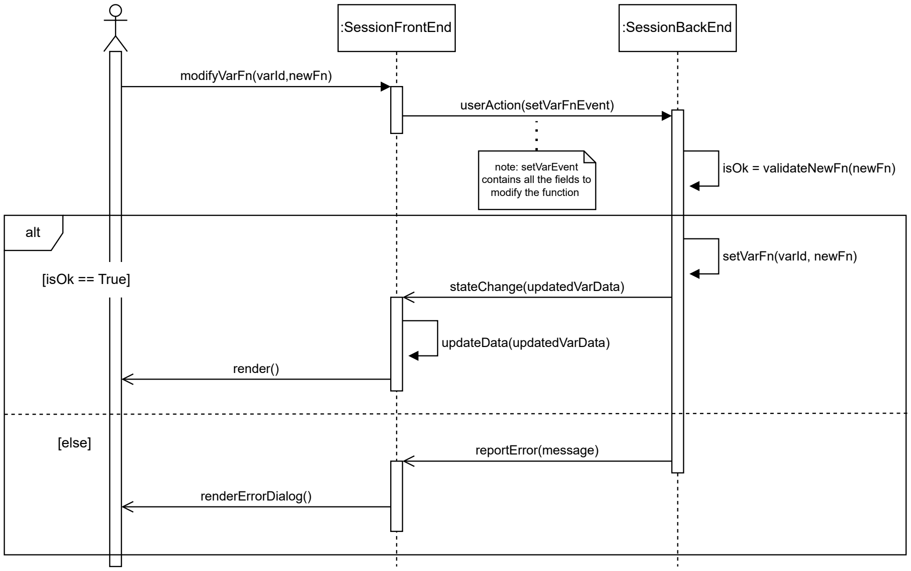

Figure 2: A high-level sequence diagram illustrating the communication between the front and back end using a simplified case of modifying a variable's update function. The front-end part of the session is represented by the *SessionFrontEnd* component, while the *SessionBackEnd* represents the back-end part of the session.

is depicted using a sequence diagram in Figure 2.

The state of the application managed on the back end is organized hierarchically. Each component in this hierarchy processes specific events and can delegate others to the appropriate components in the layer below. Events are assigned a path identifier, which directs them to the component responsible for handling them. This modular approach streamlines event processing.

The payload of *user action* events is typically minimal, usually consisting of the ID of the entity to be updated and the new user input. For instance, if the user wants to update the variable's name, we send a particular event with the variable's ID and its new name. Payloads of *state change* events can also be small (such as updates to individual variables), but they may also represent larger logical units, such as entire datasets, when necessary.

The back end also offers a mechanism for communication between sessions. While this feature is not critical for an initial release, it is designed to support future functionality as more session types are added. We call these special events *inter-session messages*. Processing such a message can trigger a response message and may also result in a state-change update for the front end. This mechanism is currently utilized when dynamically creating new inference sessions. Once an inference session is created, it sends a request to retrieve sketch data from the originating editor session and receives the data in response.

2.3.3 Undo-Redo Stack

The event-based communication system also allows us to efficiently implement *undo-redo* functionality, enabling users to revert or repeat their actions. This is achieved by maintaining two event stacks on the session's back end: an *undo* stack and a *redo* stack. Each reversible action is paired with a reverse variant, allowing actions to be undone. For example, adding a variable can be reversed by removing it.

The most recently processed event is placed at the top of the undo stack. When the user triggers the undo action, the reverse variant of the top event in the undo stack is applied, effectively undoing the last action. The event is then moved to the top of the redo stack. Similarly, when a redo action is triggered, the original variant of the top event on the redo stack is reapplied, redoing the previously undone action.

Certain events are excluded from the undo-redo mechanism. For example, "one-way" actions like exporting a model bypass the stacks altogether. Certain large events, such as importing an entire sketch, reset both stacks. The undo-redo functionality is, for now, utilized mainly within the editor session.

2.4 Editor Session

The editor session (or "sketch editor") is the part of the tool that is responsible for everything related to the process of designing a BN sketch. It is the initial session opened during the tool's startup. When the user completes the sketch editing part, they can open the inference session (described in Section 2.5) from there.

The state of the edited sketch is managed on the back end using a hierarchical structure. At the top level, the overall sketch state is divided into components such as the model, observations, and properties, with each further broken down as needed. This modular organization ensures a clear separation of responsibilities, simplifying state management, updates, and future extensions of the tool.

The tool starts with a simple initial screen that guides users to select their first action. The user can start by loading a sketch in various formats, creating a new empty sketch, or choosing a prepared example. The initial screen's design is shown in Figure 3.

After the user selects the initial action, the editor's main screen is displayed. The editor consists of several tabs where users can edit various parts of the sketch. There are the following tabs, each briefly described in its own subsection below:

- Network Editor tab
- Functions Editor tab
- Observations Editor tab
- Properties Editor tab
- Annotations Editor tab

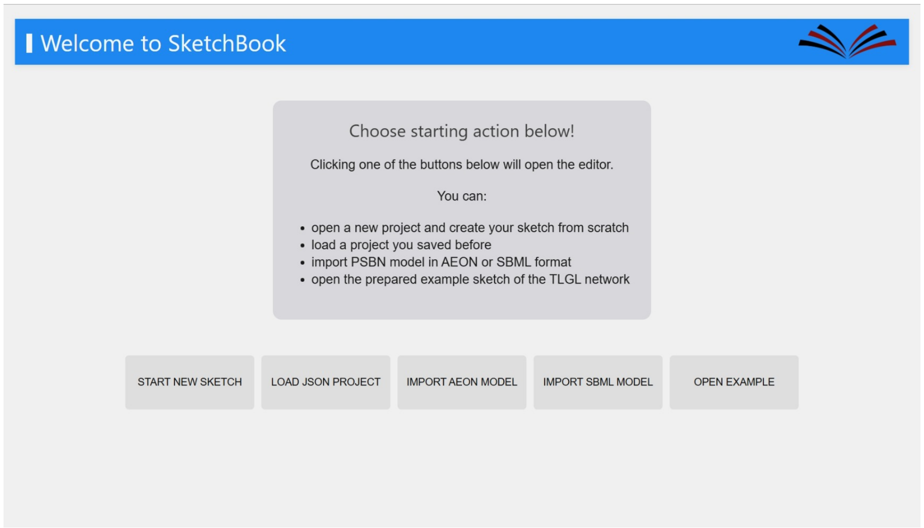

Figure 3: Initial welcome screen of the tool.

- Analysis tab

The user typically selects a tab to edit its content and then moves to another. Tabs can also be "locked," allowing two tabs to be displayed side by side. The editor session offers undo and redo functionality to facilitate revisions. Additionally, a pop-up menu in the top-left corner provides options for importing and exporting sketches in various formats.

2.4.1 Network Editor Tab

The Network Editor tab provides a graph-based editor to construct and edit the regulatory graph (where variables are nodes and regulations are edges). Users can create and edit variable nodes, adjust their positions to achieve an arbitrary layout, and select different types of regulations in terms of essentiality and monotonicity. Properties of regulations are highlighted with the edge colour and style. Clicking on nodes or edges gives the user a context menu with all relevant options. The UI of the tab is illustrated in Figure 4. Networks can be exported in JSON, AEON, or as a PNG.

2.4.2 Functions Editor Tab

The Functions Editor tab serves to set the partially specified update functions for all variables. The tab displays a summary of variables currently present in the network. For each variable, there are options to adjust all its regulators and its update function expression.

Users can also create *supplementary functions* that can then be used as part of update function expressions. These supplementary functions essentially

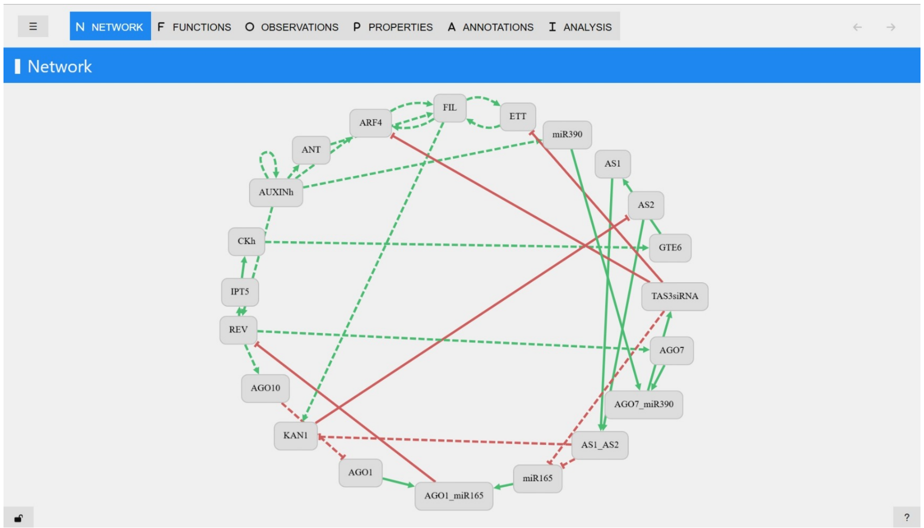

Figure 4: Interface of the Network Editor tab. Apart from the graph-based editor, this also shows the session's main navigation points. At the top, there is a pop-up menu with import/export options, a navigation bar with all the editor tabs, and the undo-redo buttons.

correspond to the function symbols in the BN sketches framework. The user can create new supplementary functions, adjust their arity and properties, and even assign them partially specified expressions. If there are many supplementary functions, users can hide their details by collapsing them for better readability. The Functions Editor is illustrated in Figure 5.

2.4.3 Observations Editor Tab

The Observations Editor tab allows the user to import or create datasets. A dataset is a collection of individual observations, where each corresponds to a single binarized measurement. Practically, each observation is an assignment to a set of model variables. The observation might assign a value to all model variables or leave some of them "empty" (this can reflect the fact that some genes were not measured or that the data quality was poor).

The datasets and their observations can be used (referenced) within the dynamic properties to integrate experimental data into constraints on the model's required behaviour. We discuss this in Section 2.4.4.

The most straightforward way to create a dataset is to simply load it from a CSV file and choose which observations to import. However, users can also create new datasets from scratch.

After creating the dataset, users can explore it and manually edit all of its components via a table-based editor. The rows of the table correspond to individual observations, while the columns correspond to the variables. Users

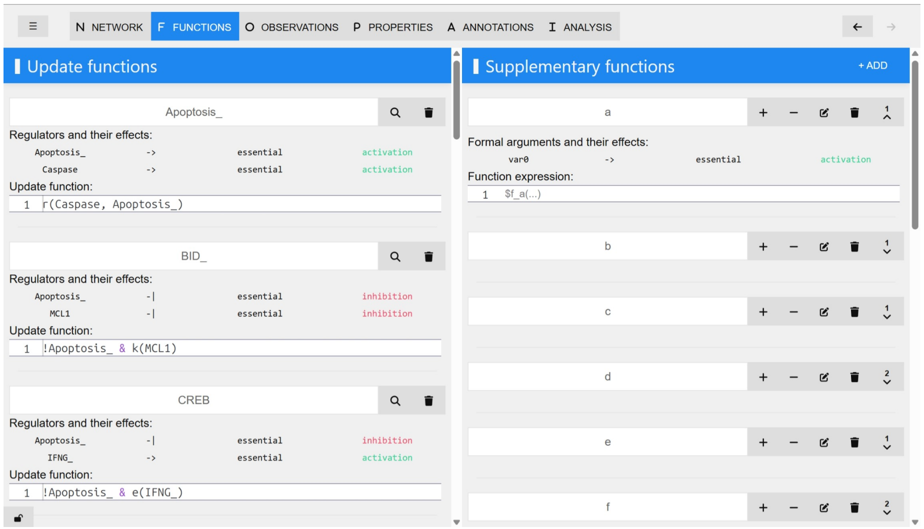

Figure 5: Interface of the Functions Editor. The variable update functions are on the left, and supplementary functions are on the right (most of them being collapsed).

can add or remove variables in the dataset and edit their names. The user can also add or remove observations and set all their values (one by one or all at once). The design for the dataset table is shown in Figure 6.

2.4.4 Properties Editor Tab

The Properties Editor serves as a way to create static and dynamic properties. When creating new properties, the tool offers several types of predefined templates from which to choose. The options labelled as *generic* allow users to write their own property in FOL or HCTL. After creating a new property, the user can set its parameters (if there are any). The interface is illustrated in Figure 7.

The set of offered templates for static properties is listed in Table 2.4.4, together with their parameters. For monotonicity properties, the user chooses from the following options – *activation*, *inhibition*, *dual* (non-monotonic), or *unknown* (can be any). For essentiality properties, the options are: *essential*, *non-essential* (has no effect), and *unknown* (could be any). Some properties are automatically derived from the regulation constraints selected in the Network Editor tab. The user can hide these properties as there may be numerous. All user-created properties can also include a FOL context formula. The properties with such context are required to hold only in situations when the context formula is satisfied.

A similar list of predefined templates for dynamic properties is given in Table 2.4.4. For properties regarding the existence of attractors, trap spaces, or fixed points, the user typically selects a dataset and its observation. This

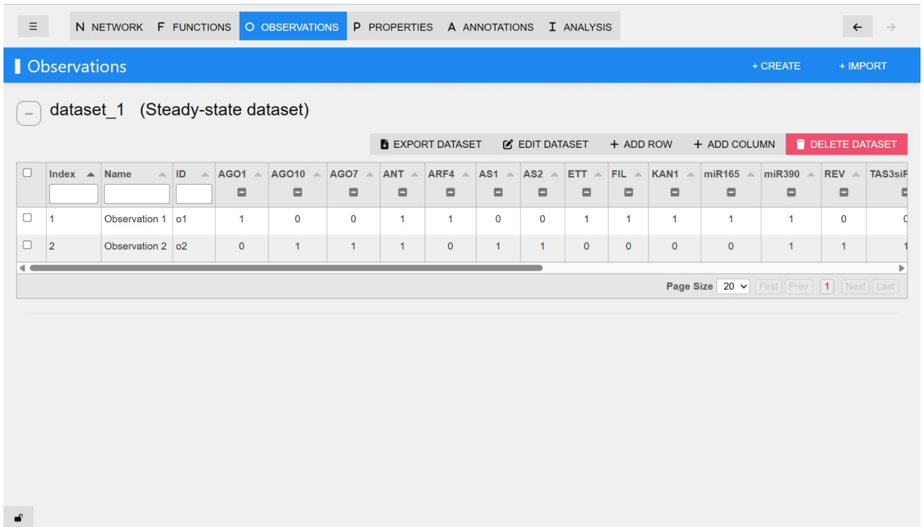

Figure 6: Interface of the table-based Observations Editor with an example dataset. Datasets can be edited directly by clicking the table or by using the edit buttons.

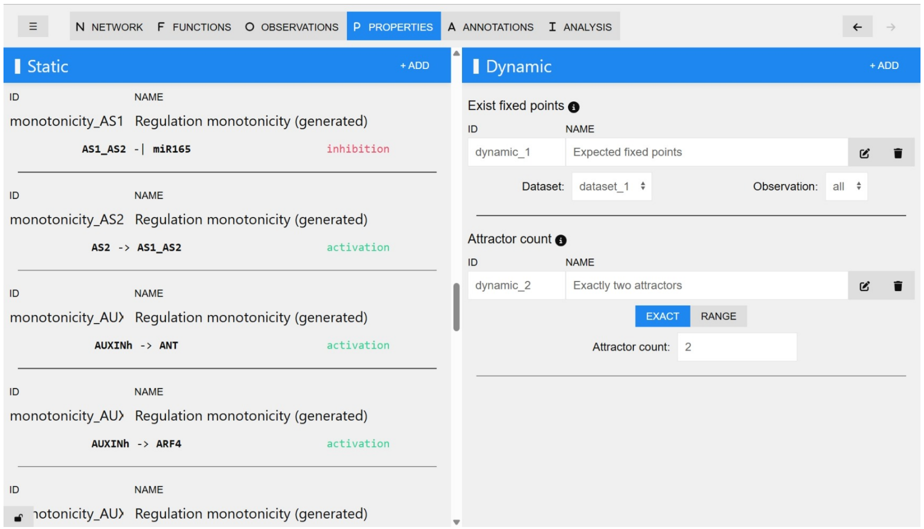

Figure 7: Interface of the Properties Editor. The static properties are on the left, the dynamic on the right. This example shows several automatically derived static properties encoding regulation monotonicity. The dynamic properties illustrate two of the possible templates, one integrating a dataset.

Template name	Parameters
Regulation essential	regulating variable, target variable, essentiality option
Regulation monotonic	regulating variable, target variable, monotonicity option
Function input essential	supplementary function, input variable, essentiality option
Function input monotonic	supplementary function, input variable, monotonicity option

Table 1: Overview of templates for static properties. Each template is listed with its parameters. Important parameters and options are discussed in Section 2.4.4.

Template name	Parameters
Attractor count	minimal count, maximal count
Exists trajectory	dataset
Exists fixed points	dataset, observation
Exists trap spaces	dataset, observation, is minimal, is non-percolable
Exists attractors	dataset, observation

Table 2: Overview of templates for dynamic properties. Each template is listed with its parameters. The templates and their options are discussed in Section 2.4.4.

observation is used to automatically generate (in the background) an HCTL formula stating: "There exists a state corresponding to the given observation that lies in an attractor (or a trap space, or fixed point)." If the observation is omitted, the formula is created for all observations in the dataset. The *trap space* property template additionally lets the user specify whether the trap space should be non-percolable (i.e., cannot be reduced through a simple percolation) or minimal. For the *attractor count* property, the user can specify the number of the model's attractors by either a single number or a range. The *trajectory* property requires the existence of a trajectory between states corresponding to subsequent observations in a dataset, and can be used to encode time series.

2.4.5 Annotations Editor Tab

Throughout the editor, users can create custom annotations for each variable, supplementary function, dataset, observation, and property. The annotations tab encompasses all these annotations and displays them in one place. The annotations are displayed in a structured way. At the top, the user can edit the annotation for the whole sketch. After that, annotations for all entities (grouped by their type) follow. The annotated entities are variables, functions, datasets, individual observations, static properties, and dynamic properties. For each

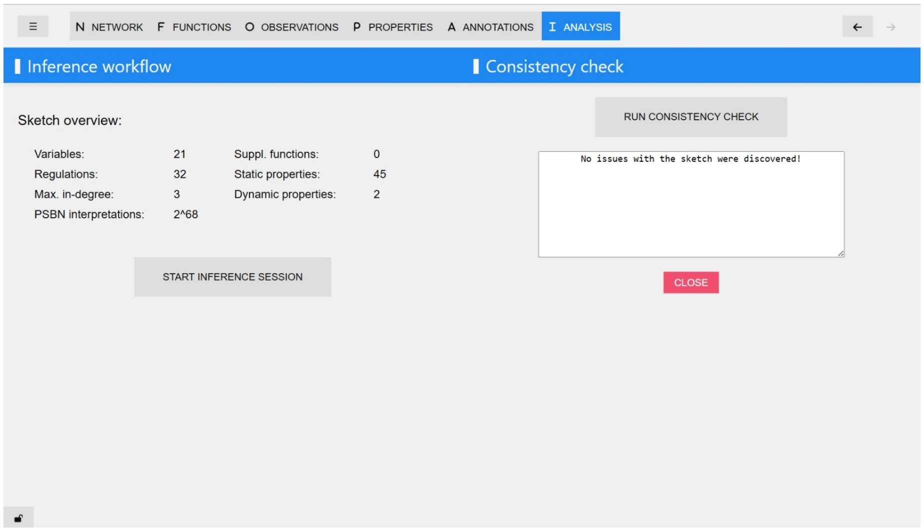

Figure 8: Interface of the Analysis tab. There is a sketch summary and the button to start the inference on the left, and the consistency check interface on the right.

entity, its ID and the annotation are shown. Datasets are shown together with their observations.

2.4.6 Analysis Tab

The analysis tab is intended as the starting point for all analysis-related sessions. Currently, its main function is to allow the user to start an inference session. In the future, all other potentially added types of analyses will also be initiated from this tab.

Additionally, the tab also provides a short summary of the sketch (number of variables, maximum node in-degree, ...) and an explicit option to run a consistency check. The consistency check performs a detailed syntactic validation of the sketch. Any potential issues with the sketch are reported to the user. This check is also automatically triggered when starting the inference, and any detected issues must be resolved before proceeding. The UI of this tab is shown in Figure 8.

2.5 Inference Session

The inference session offers a straightforward way to run the algorithms for BN inference from Section 2.2. It is initiated from the editor session, using the state of the sketch at the time of session creation as the input for inference. Since each session manages its data independently, any subsequent changes made in the editor do not affect the ongoing inference. This design allows users to open

multiple independent inference sessions simultaneously, each with different input data.

When the inference window is created, the user is presented with two options: running the full inference with all properties or performing a partial "static" inference with static properties only. The latter option is provided because evaluating static properties is typically significantly less computationally demanding than dynamic ones. This allows users to start by running this preliminary step and checking the size of the candidate set. If the candidate set is too large, they can first refine the sketch with additional static requirements before proceeding to execute the full inference.

Once the user triggers the inference, the computation begins on the back end. The algorithms run entirely asynchronously, ensuring a non-blocking execution that allows the back end to process other requests simultaneously.

During the computation, progress is continuously reported to the user. The interface displays the number of properties left to process and provides a detailed progress report. The report shows the current state of the inference and details on how the number of candidates gradually evolves.

At any point, the user can cancel and restart the computation. In the background, the ongoing computation completes its current task (such as evaluating a single property) before being terminated.

When the inference computation finishes, the user is presented with a summary of results, a progress report, and options to export the full symbolic results or sample individual admissible BNs. Figure 9 shows the design of the inference results screen.

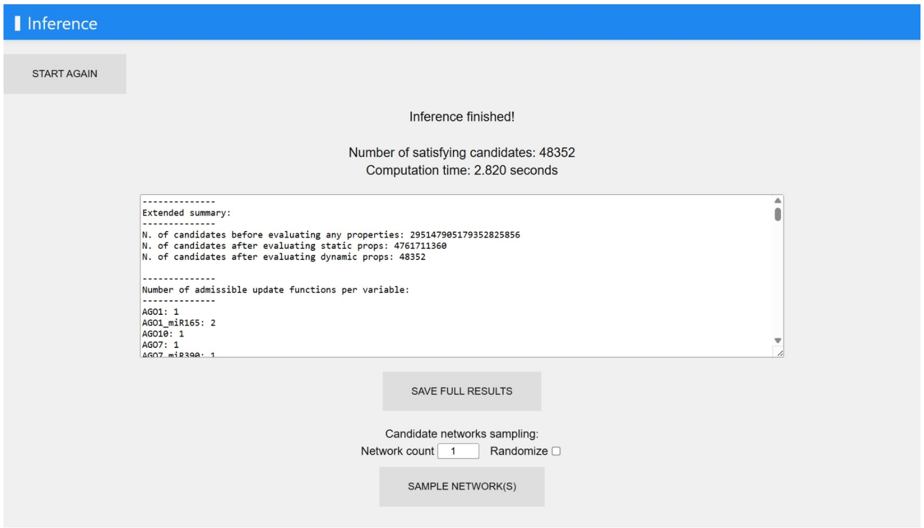

Figure 9: The UI of the inference window once the computation is finished.

The summary includes the number of admissible candidates at various stages

of the algorithm, computation times, and the number of admissible update functions per variable. The summary of admissible update functions helps users to identify which parts of the model are consistent across candidates and which are more variable, enabling them to refine the sketch accordingly.

The user can also sample the candidate networks. They can choose a number of candidates to generate and a sampling mode. We offer either a simple *deterministic* mode or a *random* mode. For the *random* sampling, users can choose a seed number for replicability. Furthermore, the user can export the symbolic representation of the whole candidate set and use it for further analysis. The results are exported in an archive that contains the following components:

- A serialized BDD encoding the set of all candidate BNs. The BDD format is compatible with the format used by the `AEON.py` library [8].
- Original sketch used as input for the inference. This is included for replicability.
- Derived parametrized model used internally during the computation. This model (also compatible with the `AEON.py` library) gives context to the BDD variables.
- Computation report encompassing all the information shown in the UI.
- List with admissible update function variants for each variable. That is, for each variable, we include each possibility for its update function that is present in some candidate BN.

3 Performance evaluation

This section provides more details on the performance evaluation that is briefly discussed in the main article. We evaluate the performance of Sketchbook on a set of complex BN sketches derived from large real-life models and synthetically generated steady-state data.

We started by selecting several real-life (fully specified) Boolean models from the BBM repository [10]. The database contains the BN models together with their influence graphs with regulation characteristics (monotonicities and essentialities). Our selection includes BNs of various sizes, ranging from 9 to 321 variables, as listed in Table 3 (this table is an extended version of the table in Fig. 1 in the main article). For each selected BN F, we compute its attractors using the algorithms in the *AEON.py* [8] library. From the unique computed attractor states, we derive a synthetic attractor dataset, emulating steady-state experimental observations.

We then prepare a partially specified model for the inference, "relaxing" F by replacing some of its update functions with function symbols (with the same arity and arguments as in the original model). This process creates a partially specified BN E, where the original network F is one of the instances of E. The PSBNs are created to capture a large degree of partial information. They contain up to 58 function symbols (including zero-arity ones), with arities up to 4, and as many as 22 *non-zero-arity* function symbols in a single model. For the three largest models, over 2^{100} BNs are consistent with the given PSBN specification and must be considered by Sketchbook during inference.

We use Sketchbook to design a model specification, combining all the components into the sketch $\mathcal{S} = (I, E, \Pi, \Omega)$. Here, I is the original influence graph of the model and E is the PSBN created as described above. Π is the set of automatically generated static properties encoding the regulatory characteristics (monotonicity and essentiality) of the original model. Finally, Ω represents the set of attractor properties derived from the prepared dataset. Particularly, we use the *Exist Attractors* property template that requires that every observation in the dataset corresponds to a state within an attractor. This property ensures that the tool checks for all kinds of asynchronous attractors, including oscillations and disordered attractors, not just fixed points.

The tool then applies the inference algorithm to compute the set of all Boolean networks consistent with the specification (sketch $\mathcal{S}$). We measure the computation time, which includes every step from reading the input to generating the entire set of all admissible solutions. After the process is complete, we also verify that the resulting ensemble of candidates contains the original network F (which is always the case by design).

Table 3 summarises the computation times and the sizes of the sets of PSBN interpretations that Sketchbook is handling at various stages of the inference algorithm for each model. The results demonstrate that Sketchbook can deal with networks of varying sizes, including those with hundreds of variables. Sketchbook can also handle a substantial degree of missing information, corresponding to a large number of potential PSBN interpretations that need to

be processed.

The computation for smaller models with a few dozen variables only takes a couple of seconds. For larger, more complex networks, the tool still completes the inference in under 10 minutes. All experiments have been performed on a standard laptop with an 11th Gen Intel i5 CPU and 16 GB RAM.

Model	No. of variables	Consistent with I, E	Consistent with I, E, Π	Consistent with I, E, Π, Ω	Time
Cell Div B [11]	9	4.1e6	6.4e4	1.4e4	0.2s
E Protein [12]	35	1.8e22	9.4e5	1008	2.5s
NSP4 [12]	60	7.5e22	1.2e6	128	2.1s
ETC [12]	84	4.7e21	2.4e8	3.1e6	370.1s
Interferon I [12]	121	1.0e31	5.3e22	6.8e5	170.7s
NSP9 [12]	252	8.5e37	3.1e12	5.5e10	28.3s
Macrophage [13]	321	2.0e31	9.9e12	7.8e11	332.2s

Table 3: Performance evaluation of SKETCHBOOK on a set of partially specified models of various sizes. The first two columns provide the model's name and variable count. The following three columns show the number of PSBN interpretations handled by SKETCHBOOK at various stages of the inference algorithm. For each sketch $S = (I, E, \Pi, \Omega)$, SKETCHBOOK starts by symbolically encoding a set of all BN candidates consistent with the plain influence graph I and PSBN E (column 3). This is essentially the initial size of the encoded solution space before considering any static or dynamic properties. The tool then refines the set, filtering interpretations consistent with static properties Π (column 4). Finally, the set is further refined to only the subset of interpretations that are also consistent with all dynamic properties in Ω (column 5). This is the size of the set of all BNs that satisfy the entire specification, which is returned by the tool. The last column reports the computation time in seconds, which includes every step from reading the input to generating the entire set of admissible solutions.

3.1 Comparison with enumerative approach

To complement the evaluation of our tool on dynamic properties that cannot be handled by existing approaches, we also provide a comparison with an enumeration-based method, represented here by BoNesis. While BoNesis employs symbolic techniques and can handle large models and parameter spaces well, it ultimately relies on enumerating or sampling individual solutions. This becomes impractical when the number of satisfying networks grows very large (which may happen, for example, when the PSBN contains function symbols of high arity, or when the amount of data is insufficient). In contrast, our approach maintains a compact symbolic representation of the entire solution space, enabling efficient exhaustive reasoning even in such cases.

This comparison is intended to highlight the conceptual and practical differences between the two paradigms rather than to serve as a direct performance benchmark. Note that BoNesis is also able to sample ensembles of diverse models, partially addressing this issue.

For this experiment, we took the two largest models from the previous section. We used the same set-up as for the experiments in the previous section, with two changes. First, to create a specification that lies within a common fragment of the two tools, we only consider simpler fixed-point attractors for dynamic properties. In particular, the derived steady-state observations are now used to specify model fixed points (instead of more complex general attractors). Second, we extend the partially specified models with additional function symbols, increasing the amount of missing information. This is feasible because evaluating fixed-point properties is significantly less computationally demanding than handling more complex attractors (enabling us to work with more complex models).

The results are summarised in Table 4. We see that SKETCHBOOK has no problem returning the set of all admissible BNs in under two minutes. BoNesis is able to enumerate ten thousand networks in a matter of seconds. However, in both cases, that is only a small fraction of the entire set of admissible networks. We use the mean computation time to estimate the time to enumerate all admissible solutions (by multiplying it by the total number of solutions). These estimates come to 199 years and more than 1.2 million years, respectively. This demonstrates the infeasibility of enumerative approaches to obtain exhaustive results in certain scenarios.

Model	No. of variables	PSBN interpretations	Total BN solutions	Sketchbook time all solutions	BoNesis time first 10k solutions	BoNesis time estimated
NSP9	252	5.8e48	3.5e13	11.2s	1.8s	6.3e9s
Macrophage	321	2.2e43	9.2e16	94.7s	4.5s	4.1e13s

Table 4: Comparison between SKETCHBOOK and enumeration-based BoNesis on large partially specified models. The first two columns provide the model's name and variable count. The third column shows the number of interpretations of the input PSBN, i.e., the number of BN candidates consistent with the plain influence graph I and PSBN E. This is essentially the initial size of the encoded solution space before considering any static or dynamic properties. The fourth column shows the size of the set of all BNs that satisfy the entire specification. Next, we report the SKETCHBOOK time (in seconds) to compute the entire set of all satisfying solutions. The last two columns report the computation time of BoNesis. We report the time to compute the first ten thousand solutions (which is just a small fraction of the entire set), and the estimated time to enumerate all solutions.

References

[1] Nikola Beneš, Luboš Brim, Ondřej Huvar, Samuel Pastva, and David Šafránek. Boolean network sketches: a unifying framework for logical model inference. *Bioinformatics*, 39(4), 2023.

[2] Daniel Kernberger and Martin Lange. Model checking for hybrid branching-time logics. *Journal of Logical and Algebraic Methods in Programming*, 110, 2020.

[3] Ondřej Huvar. SketchBook: Tool for inference of logical models of gene regulatory networks. Master's thesis, Masaryk University, Faculty of Informatics, 2025.

[4] Petr Ivičič. Frontend of the AEON Sketchbook application. Master's thesis, Masaryk University, Faculty of Informatics, 2024.

[5] Nikola Beneš, Luboš Brim, Jakub Kadlecaj, Samuel Pastva, and David Šafránek. AEON: attractor bifurcation analysis of parametrised boolean networks. In *Computer Aided Verification*, volume 12224, pages 569–581. Springer, 2020.

[6] Nikola Beneš, Luboš Brim, Ondřej Huvar, Samuel Pastva, and David Šafránek. BNClassifier: Classifying Boolean models by dynamic properties. In *Computational Methods in Systems Biology*, pages 19–26. Springer, 2024.

[7] Nikola Beneš, Luboš Brim, Ondřej Huvar, Samuel Pastva, and David Šafránek. Symbolic model checking of hybrid CTL on coloured kripke structures. In *Automated Technology for Verification and Analysis*, volume 15055 of *Lecture Notes in Computer Science*, pages 212–233. Springer, 2024.

[8] Nikola Beneš, Luboš Brim, Ondřej Huvar, Samuel Pastva, David Šafránek, and Eva Šmijáková. AEON.py: Python library for attractor analysis in asynchronous Boolean networks. *Bioinformatics*, 38(21):4978–4980, 2022.

[9] Tauri Contributors. Tauri framework: Build smaller, faster, and more secure desktop applications with rust, 2024.

[10] Samuel Pastva, David Šafránek, Nikola Beneš, Luboš Brim, and Thomas Henzinger. Repository of logically consistent real-world boolean network models. *bioRxiv*, 2023.

[11] Ismael Sánchez-Osorio, Carlos A Hernández-Martínez, and Agustino Martínez-Antonio. Modeling asymmetric cell division in caulobacter crescentus using a boolean logic approach. In *Asymmetric Cell Division in Development, Differentiation and Cancer*, pages 1–21. Springer, 2017.

[12] Marek Ostaszewski, Alexander Mazein, Marc E Gillespie, Inna Kuperstein, Anna Niarakis, Henning Hermjakob, Alexander R Pico, Egon L Willighagen, Chris T Evelo, Jan Hasenauer, et al. Covid-19 disease map, building a

computational repository of sars-cov-2 virus-host interaction mechanisms. *Scientific data*, 7(1):1–4, 2020.

[13] Sobia Raza, Kevin A Robertson, Paul A Lacaze, David Page, Anton J Enright, Peter Ghazal, and Tom C Freeman. A logic-based diagram of signalling pathways central to macrophage activation. *BMC systems biology*, 2(1):1–15, 2008.
